# Temperature-Dependent Stimulated Emission Cross-Section in Nd^3+^:YLF Crystal

**DOI:** 10.3390/ma14020431

**Published:** 2021-01-16

**Authors:** Giorgio Turri, Scott Webster, Michael Bass, Alessandra Toncelli

**Affiliations:** 1 Mathematics and Science Department, Full Sail University, 3300 University Blvd, Winter Park, FL 32792, USA; gturri@fullsail.com; 2 CREOL, The College of Optics and Photonics, University of Central Florida, 4304 Scorpius St., Orlando, FL 32816, USA; swebster@creol.ucf.edu (S.W.); bass@creol.ucf.edu (M.B.); 3 Physics Department, University of Pisa, Largo B. Pontecorvo 3, 56127 Pisa, Italy; 4 Italian National Research Council (CNR), Institute of Nanoscience (NANO), 56127 Pisa, Italy

**Keywords:** Nd:YLF, stimulated-emission cross-section, thermal effects, solid-state laser, rare-earth doped crystal, absorption, decay dynamics

## Abstract

Spectroscopic properties of neodymium-doped yttrium lithium fluoride were measured at different temperatures from 35 K to 350 K in specimens with 1 at% Nd^3+^ concentration. The absorption spectrum was measured at room temperature from 400 to 900 nm. The decay dynamics of the ^4^F_3/2_ multiplet was investigated by measuring the fluorescence lifetime as a function of the sample temperature, and the radiative decay time was derived by extrapolation to 0 K. The stimulated-emission cross-sections of the transitions from the ^4^F_3/2_ to the ^4^I_9/2_, ^4^I_11/2_, and ^4^I_13/2_ levels were obtained from the fluorescence spectrum measured at different temperatures, using the Aull–Jenssen technique. The results show consistency with most results previously published at room temperature, extending them over a broader range of temperatures. A semi-empirical formula for the magnitude of the stimulated-emission cross-section as a function of temperature in the 250 K to 350 K temperature range, is presented for the most intense transitions to the ^4^I_11/2_ and ^4^I_13/2_ levels.

## 1. Introduction

Nd-doped Yttrium Lithium Fluoride (Nd^3+^:LiYF_4_) is one of the most commonly used solid-state laser elements for its long fluorescence decay time at room temperature, weak thermal lensing, and natural birefringence [1,2,3,4].

It is usually optically pumped to the ^4^F_5/2_ level, or directly to the ^4^F_3/2_ upper laser level, by 808 nm or 880 nm wavelength light, respectively, though recently laser operations have also been achieved with 908 nm wavelength pump [5].

**Figure 1:**
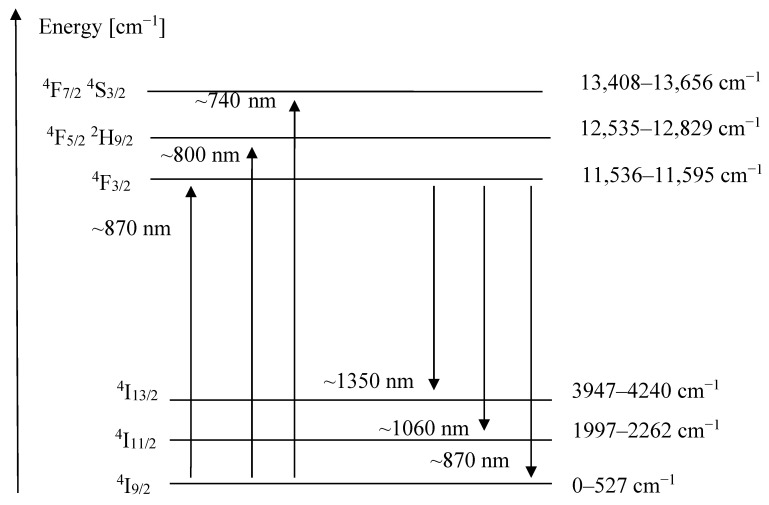
Partial energy level diagram of Nd:YLF crystal.

The three main decay channels lead to the ^4^I_13/2_, ^4^I_11/2_, and ^4^I_9/2_ levels, with the emission of light at ~1.3 μm; ~1.0 μm, and ~0.9 μm wavelengths, respectively. The most common Nd:YLF lasers, by far, operate at 1047 nm (π-polarization) and 1053 nm (σ-polarization) because of the stronger intensity, but laser operation has also been achieved for the other two final laser levels [4,6,7,8]. Figure 1 depicts the energy levels of interest for laser applications, as well as the transitions that will be investigated in this work. We are reporting the Stark multiplet energies as published by Zhang [9], other authors have reported slightly different values [10,11].

Most of the previous investigations of the spectroscopic properties of Nd:YLF have focused on the excitation and the decay of the Nd^3+ 4^F_3/2_ level at room temperature. Although the decay dynamics of that level are in general considered well understood, there is some inconsistency among the published values of the stimulated emission cross-section, especially when estimated with different techniques [12,13]. There are also disagreements about the values of the absorption coefficient, the absorption cross-section, and the general shape of the absorption spectrum in the 800–900 nm range, likely caused by low resolution, inaccurate estimates of the dopant concentration, or the presence of contaminants [13,14,15].

At higher or lower than room temperature, the thermal expansion [16], the thermal lensing [17,18], and the wavelengths shift and width [10,11] have been modeled theoretically or measured experimentally. Still, very few investigations reported the emission spectrum variation with the temperature and only over a limited temperature range [15,19]. Knowledge of the temperature dependence of the absorption, decay dynamics, and emission of a rare-earth-doped crystal or glass, carries significant information for practical applications. By controlling its operation temperature, one can tune the emission wavelength and intensity, or optimize the pump efficiency, of a solid-state laser [20,21]. Some studies have also shown the possibility of obtaining temperature-independent lasers [22,23]. For laser using birefringent or triaxial crystals as a gain medium, one can look for temperatures where the emission at different polarizations occurs with the same intensity. Following a 1969 pioneering work by Harmer [14], recently Cho et al. [15] obtained simultaneous laser emission with the same intensity, orthogonally-polarized, at 1047 nm and 1053 nm by properly cooling the Nd:YLF crystal at cryogenic temperature. Optimization of the simultaneous laser performance was accomplished by finely tuning the operating temperature of the crystal. Still, no detailed investigation of the main spectroscopic parameters (cross-section and lifetime of the observed transition) has been investigated.

In this work, we present the emission spectrum and the stimulated emission cross-section of the three main decay channels of the ^4^F_3/2_ state of Nd^3+^ in LiYF_4_, at different temperatures from ~35 K to ~350 K. Moreover, we present semi-empirical formulas that allow one to estimate the stimulated emission cross-section as a function of the sample temperature for a few selected wavelengths of possible use for laser purposes. Finally, the absorption and the emission spectra collected in this work at room temperature will be used to discuss some of the partial inconsistencies reported in the literature.

The stimulated emission cross-sections were obtained from the measured emission spectra using the Aull–Jenssen technique [24]; a detailed description of this method can be found in the investigations published by these authors for Nd:YAG [20] and Nd:YVO_4_ [25].

## 2. Materials and Methods

Two Nd:YLF samples were provided by VLOC (New Port Richey, FL, USA) and by ACMaterials (Tarpon Springs, FL, USA). Both were about 1 × 2.5 × 5 mm, with a nominal Nd^3+^ concentration of 1 at%, and were oriented with the optical c axis along the longest dimension.

The absorption spectrum was measured at room temperature using a Cary 500 spectrometer (Agilent, Santa Clara, CA, USA). The resolution of the spectrometer was set to 0.5 nm full width at half maximum (FWHM), and the wavelength was changed at 0.125 nm intervals.

The fluorescence emission spectra and lifetime measurements were performed in a pressurized helium cryostat combined with a heater and a temperature controller. In the fluorescence spectroscopy measurements, the sample was pumped to the Nd^3+ 4^F_3/2_ level by a continuous, 1 W diode laser emitting around 800 nm. The power of the pump laser was monitored during data acquisition, and the collected spectra were corrected for the pump variations, which were less than 1%. The emitted fluorescence, after polarization selection, was measured through a monochromator whose resolution was set to 0.5 nm FWHM. The transmission of the monochromator was calibrated through a tungsten-quartz halogen lamp of known intensity at all wavelengths.

The decay dynamic of the Nd^3+ 4^F_3/2_ state was investigated by pumping the sample to the Nd^3+ 4^F_3/2_ level with a pulsed optical parametric amplifier, set to generate a 4 ns FWHM pulse with energy around 100 mJ/pulse at ~808 nm and 10 Hz repetition rate. The emitted luminescence, after polarization selection, was collected by a fast germanium-detector (response time ~100 ns) and then processed by a digital oscilloscope.

## 3. Results

### 3.1. Absorption and Decay Dynamics

The absorption spectra of the two samples are very similar, though the VLOC specimen shows systematically slightly higher absorption at all wavelengths probably due to a slightly different actual doping level in the sample. As the differences are 10% or less, they will be ignored, and in the rest of this work, we will present the average of the measurements of the two samples.

**Figure 2:**
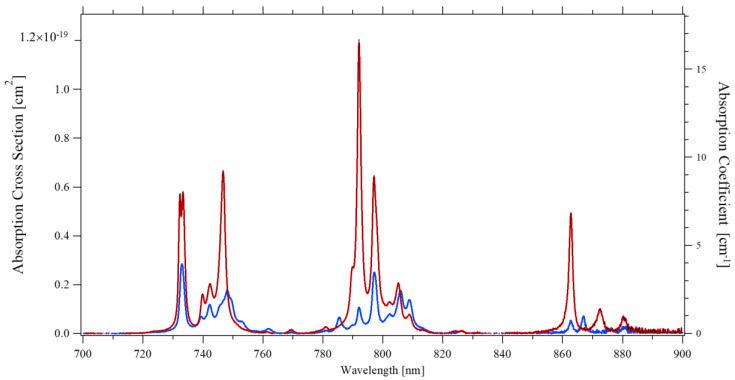
Absorption spectrum of 1 at% Nd:YLF at room temperature for π–polarized light (Red) and σ–polarized light (Blue).

A 1% Nd concentration in Nd:YLF corresponds to 1.40 × 10^20^ ions/cm^3^ as can be determined by the YLF unit-cell dimensions a = 0.5(2) nm; c = 1.09 nm [12,14,16]. Assuming this value, the absorption cross-sections as depicted in Figure 2 were obtained for σ-polarized and π-polarized light at room temperature.

The peak absorption occurs around 792 nm for π-polarized light with a cross-section of 1.2 × 10^−19^ cm^2^. For σ-polarized light, the strongest absorption in the 790 nm region occurs at 797 nm, with a cross-section of 0.25 × 10^−19^ cm^2^, though the absorption peak around 733 nm seems to have an even slightly larger cross-section of 0.28 × 10^−19^ cm^2^.

When compared with literature, our results are in agreement with Cho et al. [15], Fornasiero et al. [2], and Ryan and Beech [13] but the latter for σ-polarized light only. The π-polarized absorption spectrum by Ryan seems to have a worse resolution than ours, which would explain the difference. It is not clear what might cause the gross disagreement with the absorption spectra as published by Harmer [14], whose absorption coefficients, once rescaled to 1% dopant, are a factor 4 lower for σ-polarized light and a factor 10 lower for π-polarized light than our results.

**Figure 3:**
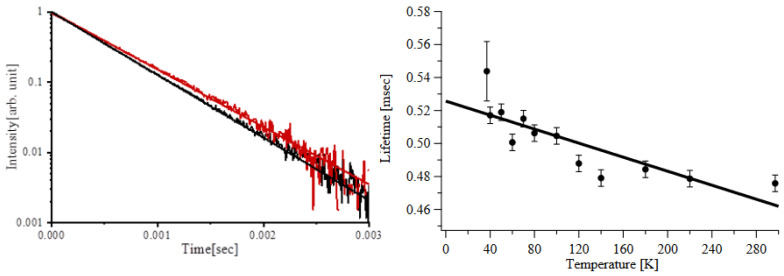
(**left**) Fluorescence decay of 1% Nd:YLF, ^4^F_3/2_ level at the temperature of 30 K (red and 300 K (black). (**right**) Measured lifetime vs. sample temperature, the continuous line is a linear best fit.

An example of a typical exponential decay from the ^4^F_3/2_ state is shown in Figure 3(Left). Figure 3(Right) depicts the fluorescence decay time as recorded at different temperatures, all of which can be accurately reproduced by a single exponential. At the pump intensities used in this investigation, we do not see a significant contribution of the higher-order effects such as excited state absorption or energy transfer upconversion, described by Chuang et al. [26] and Zuegel et al. [27]. The trend of the fluorescence lifetime versus temperature shows a constant, approximately linear, increase from the room temperature value of 476 μs toward lower temperatures; by extending the linear trend, we estimate a radiative lifetime of 530 μs. The accepted value of fluorescence lifetime for 1 at% doping concentration is ~480 μs at room temperature [1], and the estimates of the radiative lifetime range between 510 μs and 550 μs [13,14]. Therefore, both the room temperature fluorescence lifetime and radiative lifetime extrapolated at low temperatures by us are in good agreement with the published values.

### 3.2. Stimulated Emission Cross-Section

#### 3.2.1. Decay to the ^4^I_11/2_ Level

**Figure 4:**
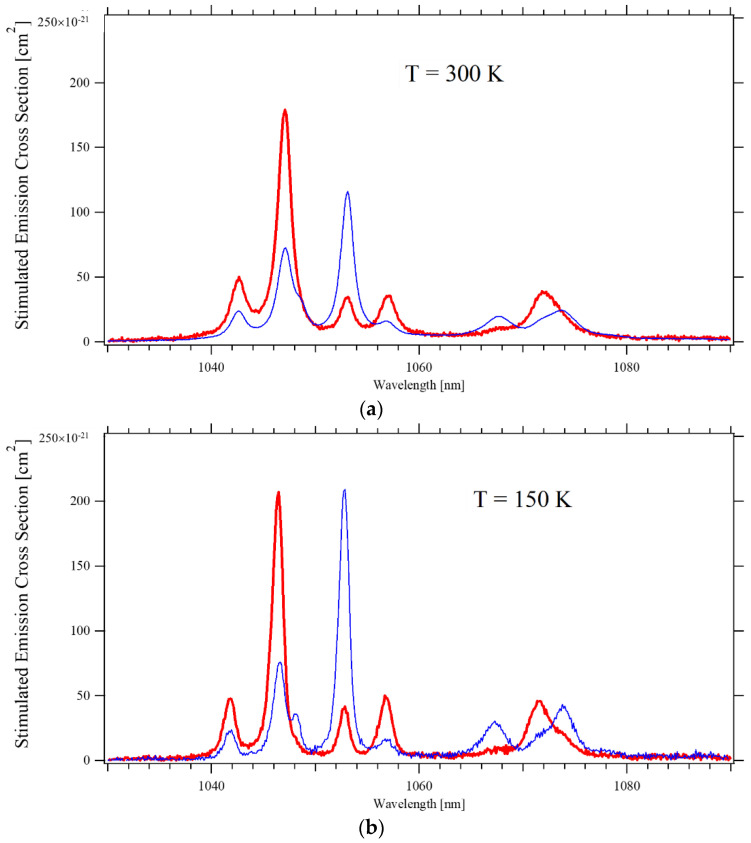

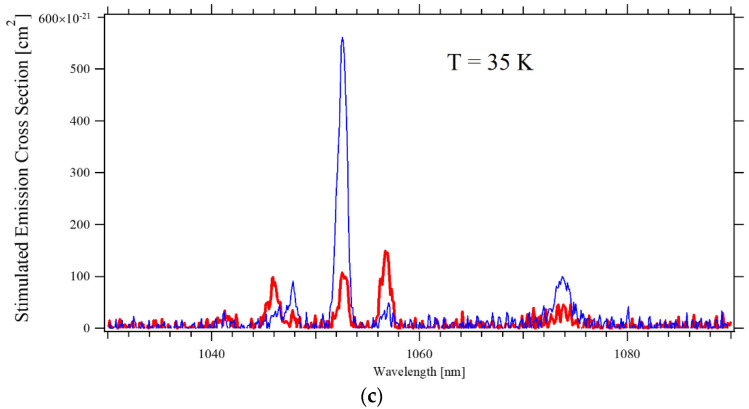
Emission cross-section of the ^4^F_3/2_ → the ^4^I_11/2_ transition for π–polarized (Red) and σ–polarized (Blue) light, at three temperatures: (**a**) T = 300 K; (**b**) T = 150 K; (**c**) T = 35 K.

Figure 4 depicts the stimulated emission cross-section of the main transition from the ^4^F_3/2_ to the ^4^I_11/2_ level at three different temperatures. The spectra measured in the two investigated samples agree within 10%, so only their average is presented.

**Figure 5:**
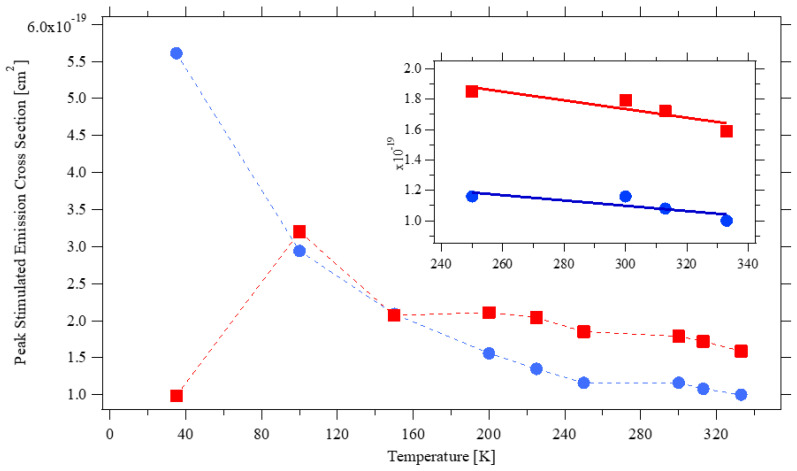
Stimulated emission cross-section for the peak emission at ~1047 nm (π-polarization, red) and at ~1053 nm (σ-polarization, blue) as a function of the temperature. The full lines in the inset are a linear best-fit in the 250 K–350 K temperature range.

At room temperature, the cross-section of the two most intense transitions at 1047 nm (π-polarization) and 1053 nm (σ-polarization) are consistent with the values measured by Pollak [1] and by Fornasiero [2], who both used the same technique as the present authors, and with Ryan and Beech [13] who instead applied the Judd–Ofelt technique. On the contrary, the estimates from Maldonado [12] based on the measurement of the laser gain are ~60% higher; this could be due to the different dopant concentration of their sample, namely 0.6 at% rather than 1 at%, or to a somehow lower accuracy of the chosen method. At temperatures ~150 K, the peak emission has the same intensity for both polarizations, consistent with Cho’s finding of the 138 K to 170 K interval (depending on the pump intensity) as the ideal temperature to achieve laser operations with the same laser output power for the two polarizations [15]. When the temperature is further lowered, the cross-section for π–polarized light drops dramatically, and at ~35 K the σ–polarized emission at 1052.6 nm dominates the spectrum. When the temperature increases, the peak emission constantly shifts toward a longer wavelength at a rate of approximately 4 nm/1000 K and 2 nm/1000 K for π-polarization and σ-polarization, respectively. Lasers are mostly operated at a temperature between 250 K to 350 K; as depicted in the inset of Figure 5, the peak intensity of the stimulated emission cross-section within that interval varies approximately linearly. By a simple best-fit regression, one obtains the semi-empirical formulas:

σ_EM_ = 2.6 × 10^−19^ (cm^2^) − 3 × 10^−22^ (cm^2^/K) × T(K) π–polarization

σ_EM_ = 1.6 × 10^−19^ (cm^2^) − 2 × 10^−22^ (cm^2^/K) × T(K) σ–polarization

A temperature increase of 10 K results in the reduction of the emission by ~2%, which is similar to what was observed in Nd:YAG [20] and about half of Nd:YVO_4_ [25]. Though difficult to estimate from Figure 6c of [21], Nd:KGW crystals seem to have a somehow lower dependence on the temperature.

#### 3.2.2. Decay to the ^4^I_13/2_ Level

**Figure 6:**
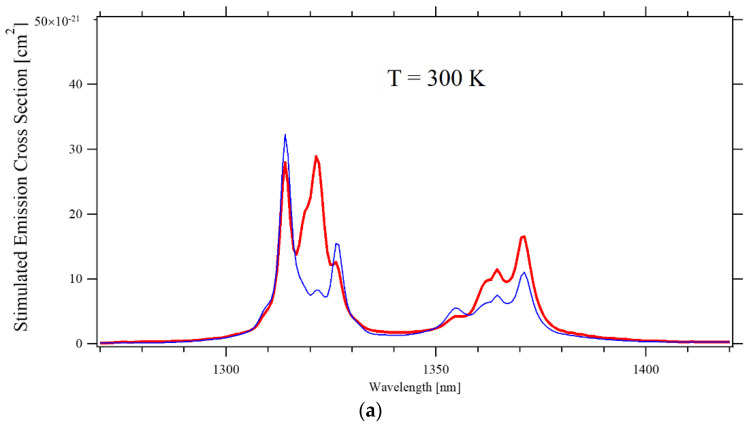

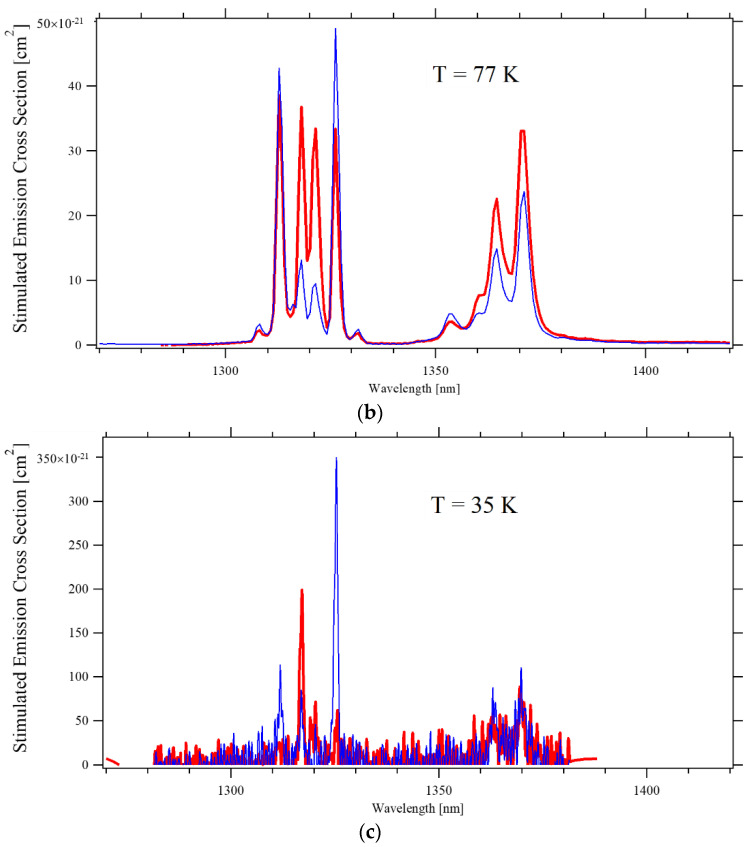
Emission cross-section of the ^4^F_3/2_ → the ^4^I_13/2_ transition for π–polarized (Red) and σ–polarized (Blue) light, at three temperatures: (**a**) T = 300 K; (**b**) T = 150 K; (**c**) T = 35 K.

Figure 6 depicts the stimulated emission cross-section for the decay of the ^4^F_3/2_ level to the ^4^I_13/2_ at three different temperatures. When compared with the measurements by Fornasero et al. at room temperature [2], the spectra from the top panel of Figure 6 have the same overall shape and intensities about 40% higher at all wavelengths. Around room temperature, the strongest emission occurs at 1314 nm for σ–polarized light and at 1314 nm and 1322 nm for π–polarized light. For laser purposes, 1314 nm seems an interesting choice since its emission cross-section is almost the same for both polarizations and remains so at least until liquid nitrogen temperatures. For that wavelength, in the 250 K to 350 K temperature range, the peak emission constantly shifts toward longer wavelength at a rate of approximately 6 nm/1000 K, and the peak emission has a linear trend well reproduced by formulas:

σ_EM_ = 7.5 × 10^−20^ (cm^2^) − 1.6 × 10^−22^ (cm^2^/K) × T(K) π–polarization

σ_EM_ = 7.5 × 10^−20^ (cm^2^) − 1.4 × 10^−22^ (cm^2^/K) × T(K) σ–polarization

A temperature increase of 10 K results in the reduction of the emission by ~5%, thus showing a stronger dependence on the temperature than the emission around 1047 nm and 1053 nm associated with the decay to the ^4^I_11/2_ level.

When the temperature approaches 35 K, the σ–polarized emission at 1317 nm and the π–polarized emission at 1325 nm rapidly increase, dominating the emission spectrum (bottom panel of Figure 6).

#### 3.2.3. Decay to the ^4^I_9/2_ Level

**Figure 7:**
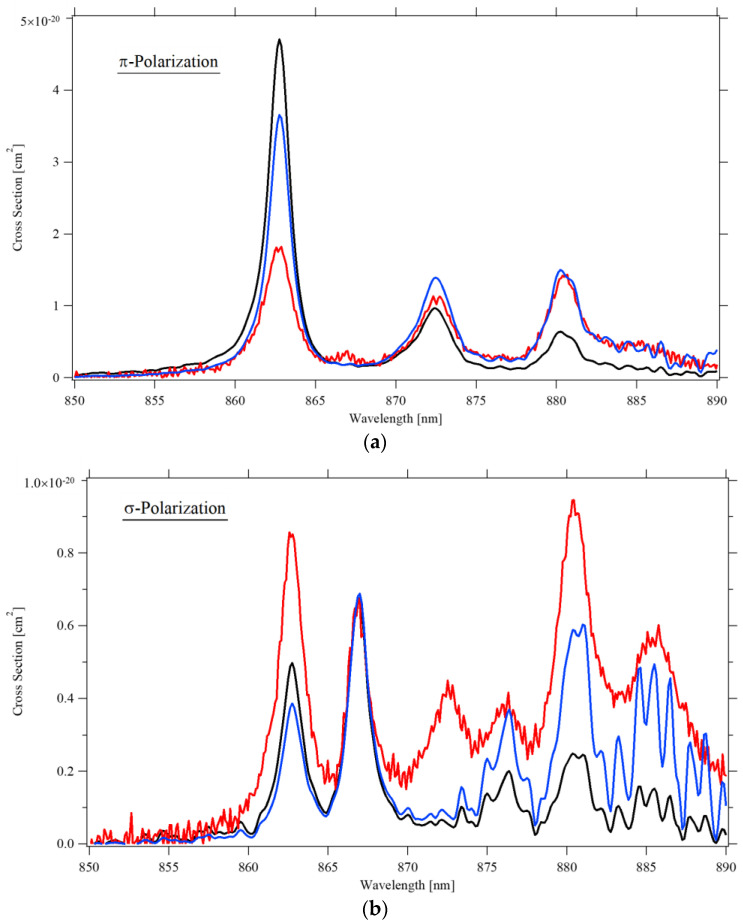
The absorption spectrum (black) and the emission spectra as obtained by Aull–Jenssen (red) and by reciprocity (blue) techniques, at 300 K: (**a**) π-polarization; (**b**) σ-polarization.

The investigation of the decay to the ^4^I_9/2_ levels is somehow more challenging. First, since the ^4^I_9/2_ is also the Nd^3+^ ground state, reabsorption is expected. To estimate the amount of reabsorption at room temperature, we applied the reciprocity method [28] to extract the stimulated-emission cross-section from the absorption cross-section. In Figure 7 the emission cross-section as obtained by reciprocity and by the Aull–Jenssen technique are compared, together with the measured absorption spectrum. For π-polarization, reabsorption only affects the peak at 863 nm, whereas for σ-polarization, the Aull–Jenssen technique seems to overestimate some of the emission peaks.

A second issue, specific to our investigation, was the low transmission of the monochromator for wavelengths shorter than 900 nm, resulting in noisy spectra, especially for temperatures below 70 K. For this reason, we will limit our investigation of the ^4^F_3/2_ → ^4^I_9/2_ transition to temperatures of 100 K or higher. It should be noticed that the contribution of the peaks in the 850–900 nm region to the total area of the emission spectrum is minor. Thus, any possible inaccuracy caused by reabsorption of excessive noise when the Aull–Jenssen technique is applied has negligible effects on the estimate of the emission cross-section of the ^4^F_3/2_ → ^4^I_11/2_ and ^4^F_3/2_ → ^4^I_13/2_ transitions.

**Figure 8:**
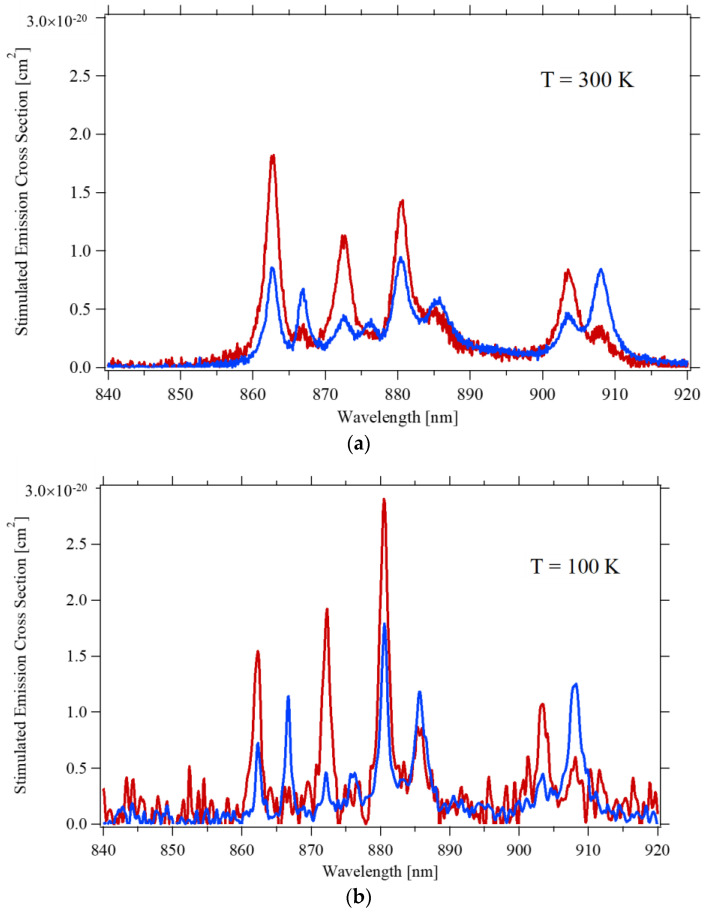
Emission cross-section of the ^4^F_3/2_ → the ^4^I_9/2_ transition for π–polarized (Red) and σ–polarized (Blue) light, at two temperatures: (**a**) T = 300 K; (**b**) T = 100 K.

Figure 8 depicts the stimulated emission cross-section for the ^4^F_3/2_ to ^4^I_9/2_ transition at room temperature and 100 K. The spectrum at room temperature is in reasonable agreement with what Ryan et al. [13] obtained through the Judd–Ofelt technique, except for the peak around 863 nm for π–polarization, and the intensity of the peaks at 903 nm and 908 nm are about 70% of the values reported by Zhang [29].

## 4. Conclusions

In this work, we investigated the absorption cross-section at room temperature and the emission lifetime and cross-section at different temperatures in the 35 K to 350 K range of 1 at% Nd:YLF crystals. The results are consistent with most previously published works and extend them over a broader temperature and/or wavelength range. In particular, we derived two sets of semi-empirical formulas that allow one to predict the stimulated emission cross-section of the peak emission at 1047 nm and 1053 nm (^4^F_3/2_ → ^4^I_11/2_ transition) and at 1314 nm (^4^F_3/2_ → ^4^I_13/2_ transition) in the 250–350 K temperature range. We also confirmed Cho’s results that at around 150 K, the peak emission of the ^4^F_3/2_ → ^4^I_11/2_ transition has the same cross-section for the π– and the σ–polarization, and showed that a similar situation occurs for the peak emission of the ^4^F_3/2_ → ^4^I_13/2_ transition, but in that case over a broad range of temperatures and with the two emissions at the same wavelength, though with a less strength.

## Data Availability

The data presented in this study are available on request from the corresponding author. The data are not publicly available because the authors do not wish to publish supplementary materials.
